# Anaerobic Digestion as a Core Technology in Addressing the Global Sanitation Crisis: Challenges and Opportunities

**DOI:** 10.1021/acs.est.3c05291

**Published:** 2023-11-13

**Authors:** Xavier Fonoll Almansa, Renata Starostka, Lutgarde Raskin, Grietje Zeeman, Francis De Los Reyes, Julie Waechter, Daniel Yeh, Tanja Radu

**Affiliations:** † Great Lakes Water Authority, Detroit, Michigan 48209, United States; ‡ Department of Civil and Environmental Engineering, University of Michigan, Ann Arbor, Michigan 48109, United States; § Wageningen University & Research, Wageningen, 6708PB, The Netherlands; ∥ Department of Civil, Construction, and Environmental Engineering, North Carolina State University, Raleigh, North Carolina 27695-7908, United States; ⊥ DigDeep, Los Angeles, California 90021, United States; # Department of Civil and Environmental Engineering, University of South Florida, Florida 33620, United States; ¶ School of Architecture, Building and Civil Engineering, Loughborough University, Loughborough LE11 3TU, United Kingdom

**Keywords:** waste(water), biogas use and emissions, recovery, WASH, challenges, stakeholder engagement, education

## Abstract

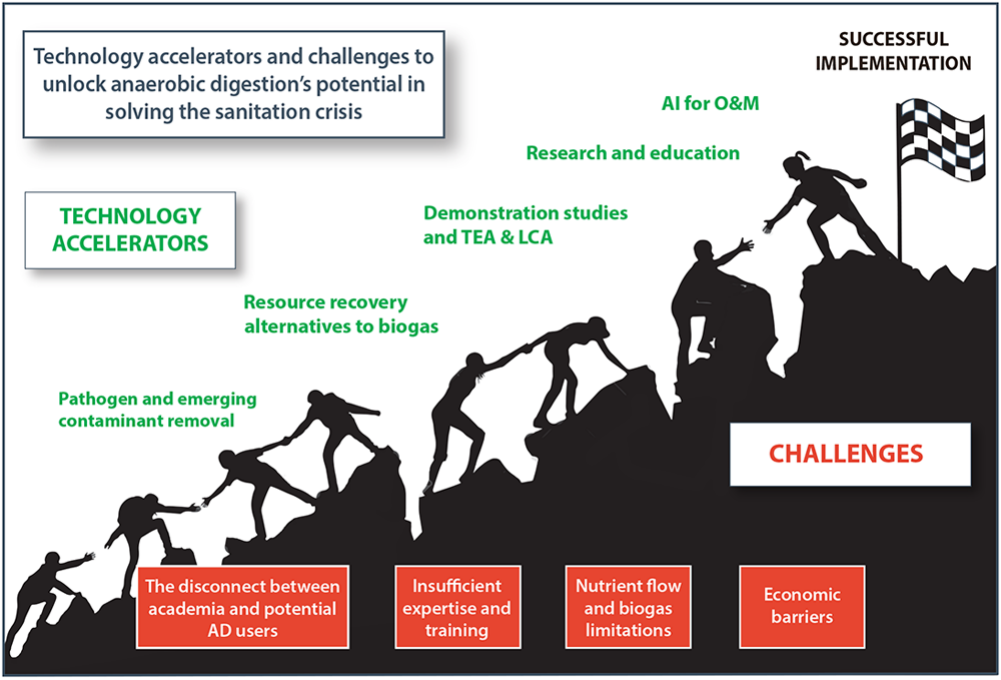

Successfully addressing the complex global sanitation problem is a massive undertaking. Anaerobic digestion (AD), coupled with post-treatment, has been identified as a promising technology to contribute to meeting this goal. It offers multiple benefits to the end users, such as the potential inactivation of pathogenic microorganisms in waste and the recovery of resources, including renewable energy and nutrients. This feature article provides an overview of the most frequently applied AD systems for decentralized communities and low- and lower-middle-income countries with an emphasis on sanitation, including technologies for which pathogen inactivation was considered during the design. Challenges to AD use are then identified, such as experience, economics, knowledge/training of personnel and users, and stakeholder analysis. Finally, accelerators for AD implementation are noted, such as the inclusion of field studies in academic journals, analysis of emerging contaminants, the use of sanitation toolboxes and life cycle assessment in design, incorporation of artificial intelligence in monitoring, and expansion of undergraduate and graduate curricula focused on Water, Sanitation, and Hygiene (WASH).

## Abstract 2

Non-technological barriers and challenges to anaerobic digestion need to be addressed to unlock its potential to help solve the sanitation crisis.

## 1 Introduction

A staggering 3.6 billion people across the globe lack safely managed sanitation services, as defined by the WHO/UNICEF Joint Monitoring Program,^1^ resulting in fecal waste being discharged into the environment without proper treatment.^2^ Among those, 1.7 billion people lack basic services; 580 million only have limited services, and 616 million use unimproved facilities. Disparities are clear, especially in rural areas where, on average, two-thirds of people lack basic services; nearly half of them live in sub-Saharan Africa.^2^

This is not just an issue in low-income countries (LICs) and lower-middle-income countries (LMICs). More than 2.2 million people in the U.S. also struggle with sanitation, lacking access to running water and a working toilet.^3^ Even among those with a (flush) toilet, ineffective management of raw sewage is common, with unsafe practices such as straight piping,^4^ cesspools, failed septic systems, and failed outhouses.^5^ For the ∼20% of households in the U.S. and other high-income countries that rely on individual onsite or small community septic systems to treat wastewater,^6^ the EPA reports that at least 10% of systems have stopped working, with some failure rates in specific communities of up to 70%.^7^

Addressing the global sanitation crisis is complex due to the interconnected impacts of culture, economics, policy, and human behavior on sanitation and its relationship with access to water.^8^ Integrated solutions are not just about providing suitable technologies but should also be framed within the enabling environment to address economic opportunities and incentives and drive behavioral change.^8^ The context-specific factors affecting these solutions include political will, the legal and regulatory framework, institutional and financial arrangements, and socio-cultural acceptance.

It is essential to consider sanitation technologies across the sanitation value chain: the user interface (the toilet), on-site storage, transport, treatment and disposal of waste, and recovery and reuse of resources. While all these links are important, a key component is the availability of waste treatment and resource recovery technologies tailored to specific communities’ needs. This means finding ways to inactivate pathogens in feces and urine or fecal sludge and, when advantageous, using fecal sludge as a substrate for resource recovery in effective, resilient, and sustainable ways in the context of underserved communities. This feature article highlights anaerobic technologies as one of the promising approaches to removing pathogens from waste and converting waste into resources in underserved communities in LICs and LMICs and communities in high-income countries not served by centralized sanitation infrastructure. While the entire sanitation chain, including the appropriate collection and transportation of the waste,^9^ should be considered in lowering public health risks, this feature article focuses only on the sanitation aspects of AD as a technology. We note that pathogen transmission can still occur during waste handling even if AD is implemented and effectively inactivates pathogens.

Decentralized or distributed anaerobic systems, coupled with subsequent resource recovery technologies, have great potential to improve sanitation in underserved communities. Depending on local conditions and constraints, decentralized anaerobic systems can be developed for individual households (one or more people occupying a housing unit) or communities (several households using the same pit latrine or community-scale transport and treatment/recovery) to fit the context. The benefits of anaerobic technologies are closely linked to meeting the Sustainable Development Goals such as Clean Water and Sanitation (SDG6), Good Health and Well-Being (SDG3) through improved water quality with reduced health risks, Gender Equality (SDG5) by reducing the need for women to manage water procurement, sanitation system maintenance, and energy, Zero Hunger (SDG2) by increasing sustainable fertilizer production, and Climate Action (SDG13) by producing renewable heat and fuel. In this feature article, we summarize the discussion of participants of the Workshop “Anaerobic Digestion, a Technology to Help Solve Water, Sanitation, and Hygiene (WASH) Concerns in Resource-constrained Communities” at the 17^th^ International Water Association Conference on Anaerobic Digestion (Ann Arbor, Michigan, June 17–22, 2022). Further analysis and discussion are provided to cover the potential, challenges, and future of anaerobic systems in addressing the sanitation challenge.

## 2 Anaerobic Digestion as a Sanitation Technology for Decentralized Communities and Low- and Lower-Middle-Income Countries

While there are many sanitation options to treat wastes, AD provides a unique opportunity to combine on-site waste treatment, pathogen inactivation, and resource recovery, which makes AD effective and desirable in small-scale applications. Biogas, which typically contains 60% methane and 40% carbon dioxide, can be recovered for heat and electricity production through combined heat and power, cooking, and lighting. Using biogas for cooking enables savings in firewood/fossil fuel use and prevents indoor air pollution by reducing incomplete combustion of firewood/fuels. Respiratory tract infections linked to indoor air pollution due to incomplete combustion account for 1.45 million deaths every year.^10−12^ Collecting biogas, which otherwise would be released during the uncontrolled anaerobic decomposition of organic wastes, also reduces the amount of methane that escapes to the atmosphere. AD also provides a nutrient-rich liquid effluent and organic-rich biosolids (sludge or digestate). The nutrients can be concentrated via methods such as struvite precipitation and ammonia stripping.^13^ The liquid effluent or its recovered nitrogen and phosphorus and the organic sludge can be used in agriculture for food production, a benefit in locations where access to chemical fertilizers is limited because of availability or cost.

### 2.1 Overview of Small-Scale and Decentralized AD Systems

AD has been widely implemented in resource-constrained environments. Single-household anaerobic digesters are increasingly used around the world. For example, China had 41.8 million household-scale biogas plants in 2014 with livestock waste, domestic sewage, and agricultural wastes as the substrates.^14^ India constructed over 90 000 family-size biogas digesters between 2017 and 2021.^15−18^ Programs in Nepal, China, and Vietnam have provided financial incentives to households that connect their toilet to an AD system. The nonprofit organization SNV has installed almost 600 000 digesters in resource-constrained settings across Latin America, Asia, and Africa.^19^

Various types of AD technologies have been implemented widely in LICs and LMICs. Common household AD systems in LMIC include the fixed dome digester, the floating drum and the tubular or plug flow digester with the HomeBiogas system being a new addition to the market.^20,21^ Although usually not considered AD technology, septic tanks are anaerobic treatment systems that are widely used in rural areas, unincorporated and underbounded urban environments,^22^ or households too far from centralized wastewater treatment systems to connect to sewers.^23^ In a septic tank, suspended solids in the influent waste stream settle to the bottom of the tank to allow for biodegradation. The limited contact between settled solids and liquid, and the lack of mixing results in a low removal of dissolved organics.^24^ Septic tank effluents are discharged into drain fields for subsoil infiltration to provide nitrification/denitrification, phosphorus sorption, and pathogenic organism removal through attenuation. However, high land requirements for drain fields often become design constraints. Due to poor conditions and maintenance, septic tanks are often responsible for environmental pollution and the spread of pathogens.^25,26^ Moreover, biogas is typically not collected, resulting in fugitive methane emissions that contribute to climate change.

Another common system is a biogas latrine, which connects community on-site toilet blocks to an anaerobic reactor for biogas production.^27^ Biogas is used in a separate room in the same building for cooking. A more developed biogas latrine is the Sulabh digester, used in over 1 million households and 3000 community toilets in India.^28^ The Sulabh digester combines a pit latrine with a sand filter, aeration tank, and carbon filter for the effluent after biogas is collected in the first chamber to produce biogas and high-quality effluent.^29^

An upflow anaerobic sludge blanket-septic tank (UASB-ST) is a modification of a conventional septic tank that operates in an upflow mode, resulting in both improved (physical) removal of suspended solids and bioconversion of dissolved organics.^24^ The height of the sludge bed increases with time, similar to a conventional septic tank. Biogas is collected through a three-phase separator as in a classic UASB reactor.^30^ Although not yet widely used, UASB-STs are seen as an attractive alternative to the commonly used conventional septic tanks. There are reports of the application of UASB-ST in Indonesia^31^ and in a small community in Palestine.^32^

The Decentralized Wastewater Treatment Solutions (DEWATS) is a widely deployed sanitation system, which has been implemented at >2700 locations worldwide and serves a total of ∼1 M people.^33^ DEWATS includes a chain of physical and biological treatment technologies such as sedimentation, flotation, and anaerobic and aerobic treatment, with AD technology as its core. Commonly applied AD systems within DEWATS are the Anaerobic Baffled Reactor (ABR) and the Anaerobic Filter.^33^ Daily per capita biogas production averaged 20 L for seven DEWATS systems deployed in Indonesia and India.^34^

Application of community on-site treatment of sanitation waste is not always possible, especially in high-density urban areas. Alternatively, fecal matter from households can be collected and transported (by truck) for subsequent treatment and recovery in an AD treatment and recovery system outside the community. An example is the Safisana plant, which opened in 2017 in Ashaiman, located in the Greater Accra Area, Ghana.^35^ In addition to fecal sludge, agro-industrial waste is collected, transported, and added to the AD system for higher biogas production. Biogas is used for electricity production and sold to local electricity companies. Each plant can produce 600 MWh and 286,000 kg of organic fertilizer per year.^36^

The above-mentioned systems (fixed dome digester, floating drum digester, tubular/plug flow digester, septic tanks, biogas latrines, and DEWATS) can transform organic wastes into biogas which is mostly used for cooking while improving waste management practices. However, even though pathogens levels in the effluent are frequently lower than in the influent, AD systems installed in LICs and LMICs are not always designed for the pathogen load they receive, especially when AD is operated at temperatures at or below the mesophilic range or at short retention times, resulting in an effluent that is not safe to manage.^9,37−40^ For example, analysis of the effluent of different DEWATS configurations in Java (Indonesia) indicated high levels of fecal coliforms that posed public health risks.^41^ A study in Ethiopia found that the levels of *E. coli*, coliforms, and *Enterococci* in the effluent of four floating drums were above the levels that the EPA considers safe for disposal.^9^ Future policies will likely be more stringent and require lower values.^42^ Thus, it is crucial to consider pathogen inactivation when designing and implementing AD at a small scale. The following section provides examples of AD-containing technologies that have incorporated pathogen inactivation into their design.

### 2.2 Pathogen Inactivation Technologies with AD Component

#### 2.2.1 Anaerobic Digestion Pasteurization Latrine

An anaerobic digestion pasteurization latrine (ADPL) consists of a toilet (with approximately 1 L of water used for flushing/cleaning) and a plastic prefabricated latrine slab built on top of a plastic floating dome digester (working volume of 2.5–2.7 m^3^). The digester is connected to a pasteurization system that uses biogas as the fuel for heating to sanitize the digester effluent and make it suitable for application in agriculture.^43^ Forbis-Stokes et al.^43^ studied ADPL systems in two residential areas in Kenya with 17 and 35 residents. During testing, the ADPL removed 85–89% of the COD and achieved greater than 5 log reduction of fecal coliforms (to nondetectable levels), making the ADPL a feasible alternative for on-site sanitation by providing effective control of fecal pathogens before effluent reuse and without external energy input. Nonetheless, the system presented some challenges due to maintenance issues. For example, the temperature for pasteurization was sometimes not achieved due to accumulation of solids in the heater and the system had to be monitored to avoid corrosion of the burners for pasteurization due to H_2_S.

#### 2.2.2 NEWgenerator

The NEWgenerator is a scalable modular sanitation technology for onsite wastewater treatment capable of meeting stringent discharge or reuse criteria.^44^ The current design of the NEWgenerator 100 can support 60+ users (300 uses/day), and it has been tested in the field in southern India and South Africa.^44,45^ The automated, solar-powered system is contained in a mini shipping container (footprint of 1.9 m by 2.4 m). It consists of a bar screen for trash removal, an underground equalization tank, an anaerobic baffled reactor with an external ultrafiltration membrane, a nutrient capture system to remove nitrogen through ion exchange and activated carbon, and a final electro-chlorination system that uses a NaCl brine to produce chlorine gas for pathogen inactivation. The most recent field trial was performed in South Africa in an informal settlement for 1.5 years. The system treated black water and yellow water and produced an aqueous stream for discharge and reuse (e.g., toilet flushing and irrigation for onsite agriculture).^44^ While detected in the influent, pathogens such as helminth ova or *E. coli* were never detected in the effluent of the NEWgenerator during the duration of the study due to the ultrafiltration membrane and the chlorination system. Protozoa and viruses (part of the requirements of the ISO 30500 for safe sanitation),^46^ were not tested, but the ultrafiltration membrane and chlorination system are expected to be effective against these pathogens as well. The system did not present major challenges related to implementation and operation, and the maintenance required was chemical cleaning of the membrane once a year. A recent study of the economic viability of the NEWgenerator indicated very positive results.^47^ Nonetheless, the system is still under examination to optimize the regeneration procedure by using less chemicals and shortening the system downtime.

## 3 Challenges in the Use of Anaerobic Digestion in Sanitation

While AD is a mature technology, implementing decentralized AD systems faces a range of challenges,^48^ which can cause technology failure or abandonment. In Africa alone, there are hundreds of failed and abandoned biogas projects.^48^ Addressing these challenges is crucial. These challenges tend to be context-specific and can include limited knowledge/training and engagement of the stakeholders and users, variable availability and seasonality of wastes, limited resources available for the commercialization of the products, unfavorable environmental conditions (such as low temperature), and limited space for installation. However, AD is a flexible technology that can be widely implemented if adapted to the context and with proper training for those operating and maintaining the system.

### 3.1 The Need for Training

For on-site AD sanitation systems serving nonsewered communities, it is most logical to only use substrates produced in the community, such as feces, urine, and kitchen/food waste. The complexity of AD treating various types of wastes (separately or together through codigestion) and generating products for different applications requires proper education and training of builders, operators, and users. Hewitt et al.^49^ report that apart from poor design and construction, the most important causes of reactor abandonment in Tanzania include lack of training for operators/users, poor reactor feeding practices, and issues with operation and maintenance. Similarly, Parawira^50^ cited lack of support and lack of knowledge as pivotal reasons for the poor effectiveness and the abandonment of biogas digesters in sub-Saharan Africa, eventually deterring technology adoption. However, Mutai et al.^27^ showed that comprehensive training of builders and users on construction, operation, and maintenance increased the performance of biogas latrines in Nairobi. Thus, the business case should include not only a budget for operation and maintenance but also training in proper maintenance procedures specific to the system and tailored to the characteristics of the substrates used. For example, the NEWgenerator was operated and maintained by a local engineer who was trained by the team installing the system.^44^ Additional training could be aided further by the development of machine learning tools that monitor digesters (see Section 4.5).

### 3.2 Economic Challenges

For AD to succeed as a sanitation technology, it should be economically viable, which may depend on the resources produced. Therefore, economic challenges are not only related to the high cost of the installed technologies and their maintenance, but also to the low price of AD products, and the competition of biogas with cheaper fuel sources. The uptake of AD technology at the decentralized level is often the result of governmental incentives and programs, making implementation more affordable for the general population. However, despite government subsidies, there are few reports of successful adoption in India.^51,52^ Indian banks and Micro Finance Institutions (MFIs) offer loans to farmers to help with the initial cost, but the poorest households struggle to access easy credit to help with installation costs.^52,53^ This economic challenge can be addressed by income-proportionate user fee distribution, where those with higher incomes and higher wastewater production pay more for a community-sharing decentralized system.^41^ The number of users is another important aspect to consider. For example, if the NEWgenerator is designed for 100 users and used by only 50 users, the cost per capita would double; conversely, if there were 300 users, the cost per capita would be reduced by 20%.^47^

Although decentralized systems are sometimes not considered financially viable, economic losses due to lack of sanitation can be much higher than the investment needed to provide a decentralized system to people without access to sanitation.^41,54,55^ For example, Kerstens et al.^41^ performed an evaluation of DEWATS in Java (Indonesia) and concluded that government investments would have substantial economic benefits. Specifically, they determined that the cost of the lack of sanitation practices for 43% of the Indonesian population was 6.5 × 10^9^ USD/year and the gains after improving sanitation would be 5 × 10^9^ USD/year.

### 3.3 Matching Stakeholder Needs and Preferences to Potential AD Products

A detailed and careful analysis of stakeholder needs, preferences, limits, and strengths along the whole sanitation chain is needed before designing and implementing any AD project. For example, resource recovery must be assessed holistically. Aiming for both maximizing biogas and full nutrient recovery at the household and community level can make the technology more expensive and complex, especially if the amount or concentration of the products obtained is too low to justify further processing. For example, recovering nitrogen as a fertilizer source from the effluent of a NEWgenerator serving 100 users would require additional purification steps and would decrease capita costs by only 2%.^47^ If resource recovery is the aim (e.g., at a city-wide level), then a thorough assessment of the business case for the products is needed. For example, large cities may not be able to use all recovered fertilizers within the city limits. To lower transportation costs, liquid fertilizer products, such as pasteurized digestate, can be used within the city, while transporting dry fertilizer products, such as dry struvite products, to agricultural fields outside the city may be more cost-effective.^56^

Combined heat and power units for generating electricity and heat from biogas can be costly at the household or community level, which limits the use of biogas to fuel cook stoves or small heaters. While most AD projects at the centralized level are implemented to safely manage waste and produce biogas, communities may choose decentralized AD to manage waste to reduce odor problems or inactivate pathogens. Insisting on biogas recovery and use in every scenario can lead to project failure and abandonment.^48^ Biogas utilization adds maintenance needs, and if the supply of biogas is neither constant nor sufficient for its intended use (e.g., slow cooking for long periods), it can lead the user to become disinterested in the technology.^49^ When biogas utilization is not justified, flaring should be required (in a safe manner) to avoid a negative impact on greenhouse gas emissions.

Selecting an appropriate treatment/recovery technology depends on the collection and transport system, type of toilet (dilution) and sewer, waste availability (other than feces and urine, e.g., kitchen waste), capacity for behavioral change, stakeholder analysis, climate/ambient temperature, and reuse possibilities. A strategy for waste transport or product removal may be necessary. Firmansyah et al.^57^ offer a methodological approach, supporting urban planning and decision-making in selecting more sustainable sanitation using AD.

## 4 Refocusing Research and Education

### 4.1 Inactivation of Pathogens and Indicator Organisms

An important benefit of AD is the ability to inactivate pathogens and indicator organisms (PIO). Research on the sanitation aspect of AD is crucial to achieving the sanitation goal of protecting public health. Research has focused on a variety of chemical (e.g., pH, ammonia, volatile fatty acids), physical (e.g., temperature, retention time, moisture content), and biological (e.g., competition, predation) approaches to inactivate PIO during AD.^58−65^ A recent approach is the use of chain elongation, a process aiming to produce medium-chain fatty acids as a more profitable product than biogas, to inactivate PIO.^66^ It is known that pathogen inactivation in AD systems at mesophilic or psychrophilic conditions is generally not sufficient. Applying a thermophilic (55 °C) UASB to blackwater treatment adequately removes pathogenic indicators while achieving the same methanisation and COD removal as with mesophilic treatment.^62^ Forbis-Stokes et al.^43^ used the biogas generated during digestion to pasteurize the digestate through a heat exchanger and they were able to reduce fecal coliforms to nondetectable levels even though average ambient temperatures in the AD reactor ranged from 12 to 22 °C. Another alternative approach is to utilize a hybrid process such as the anaerobic membrane bioreactor, in which AD is coupled with membrane filtration and other downstream processes to achieve high log reduction of PIO.^44^

### 4.2 Field Studies

Field studies are not commonly described in scientific literature and are often difficult to publish due to the lack of replication, perceived rigor, and scientific novelty. The inactivation of PIO during AD has mostly been studied under highly controlled laboratory conditions, where key stressors (e.g., low pH and high temperature) and feedstock characteristics were held constant.^61,63,66−68^ When AD is studied at a pilot or demonstration scale, the process conditions can fluctuate due to challenges with operation and maintenance or fluctuating environmental conditions. Collecting consistent data can be challenging because of issues such as a lack of sufficient volume of water to properly flush black water to the digester, changing waste characteristics, drastic temperature variations, in-field analytical limitations, and corrosion due to H_2_S in biogas.^43,66^ Despite these difficulties, publishing field studies is critical to identifying and solving real-world problems, learning from mistakes, and continuously developing a technology that works in decentralized and resource-constrained environments. Scientific journals should become more receptive to publications of field experiments performed under real-life conditions. Publishing studies discussing process failure could be the best way to avoid repeating bad practices.^48^

### 4.3 Consideration of the Emerging Contaminants

The digestate of AD-treated black wastewater also contains emerging contaminants, such as pharmaceuticals, perfluoroalkyl substances (PFAS), hormones, and personal care products.^13,69−77^ These contaminants can harm environmental and human health by bioaccumulating in soils, organisms, and crops. Reusing AD digestate in agriculture can pose public health risks through possible bioaccumulation of residues up the food chain. While AD can remove some of these emerging contaminants (e.g., ibuprofen, naproxen, Androstenedione),^78^ it does not remove all of them and the effluent might still require additional treatment, such as biochar adsorption, composting, and physical and chemical treatment.^69,71,72,77^

### 4.4 Sanitation Toolboxes and Life Cycle Assessment

Tools such as the Fecal Sludge Management Toolbox^79^ or EAWAG’s Compendium of Sanitation Systems and Technologies^80^ allow communities to engage with sanitation projects and select system components. A good design tool should incorporate geographic challenges (e.g., soil type), infrastructure challenges (e.g., access to water flow or electricity), social challenges (e.g., preferences for the human interface), scientific instructional videos (sizing an AD system, nutrient recovery benefits and options, proper sanitation practices), and region-specific life cycle assessment (LCA). The Quantitative Sustainable Design tool (QSDsan) addresses both LCA and design aspects of sanitation and resource recovery systems.^81^ This application could be extended with sensor-based monitoring and simple tests throughout the lifespan of the technology.

Several studies have performed LCA to compare sanitation designs (e.g., toilet designs in India,^82^ EcoSan versus conventional sanitation,^83^ source separation versus conventional systems,^84−86^ and full waste management system comparisons in South Africa^87^ and Egypt^88^). When performing an LCA, it is important to consider all potentially relevant aspects, such as toilet and transport design, methane losses, construction costs, and the entire sanitation value chain. In addition, it is critical to account for methane losses and greenhouse gas emission analysis^89−91^ since anaerobic digesters at the household or decentralized scale can present fugitive methane emissions in pipe connections, pressure-relief valves, and during digestate storage. Also, in the absence of biogas storage tanks, farmers sometimes discharge surplus biogas to reduce the risk of explosion.^92^ Some studies that considered fugitive methane emissions in their LCA concluded that, even with fugitive emissions, households with AD have 48% lower greenhouse gas emissions than households without digesters.^93^ Nevertheless, it is critical to ensure that the systems do not have leaks and avoid the installation of systems without a capture system for biogas. While being in line with the sanitation goals of SDG6 such systems may negatively impact other sustainability goals such as SDG13, which is focused on combatting climate change.

### 4.5 Operation and Maintenance with Artificial Intelligence

Field studies have shown that maintaining optimal operation by monitoring basic stability parameters at the community or household scale can be challenging because of the unavailability of analytical laboratories or a scientific workforce. New research efforts should focus on the use of artificial intelligence to develop analytical tools that can be applied in the field and are user-friendly (i.e., mobile phone apps and easy-to-use in-field test kits).^94^ Using field data from an onsite treatment system, Shyu et al.^95^ applied machine learning algorithms to develop “soft sensors” for predicting water quality parameters conventionally measured offline in laboratories (e.g., COD and TSS), using low-cost inline sensors such as pH, color, and turbidity. The use of such tools can be improved with better artificial intelligence and monitoring capabilities. In addition, the use of low-cost remote monitoring platforms offers an exciting prospect for decentralized community digesters. For example, such systems would enable automated monitoring of digesters’ stability and would be equipped with alarms to sound if predefined threshold levels for monitored parameters were exceeded.^96^ This field of research could lead to the development of AD systems that are easier to monitor and operate, addressing one of the implementation challenges (also see Section 3.1).

### 4.6 WASH Curricula

Proper workforce training is crucial for the successful application of AD to tackle sanitation challenges. While there are master study programs focusing on WASH-related topics, WASH is rarely included in undergraduate curricula. WASH-related topics are sometimes introduced at the undergraduate level, mostly to highlight the current WASH challenge, and in the context of SDG6. However, design for WASH applications is rarely taught as part of traditional Environmental Engineering programs. The opportunity is often missed to include WASH in courses such as wastewater treatment/engineering, which mostly focus on the conventional treatment options used in the Global North; the numerous real-world, practical, and contextual issues that communities in LICs and LMICs face are overlooked. The inclusion of WASH in traditional undergraduate Environmental Engineering curricula presents an opportunity to educate and inspire new generations of WASH-aware engineers. This requires that educators themselves connect and learn from the WASH community. Just as the inclusion of sustainability and green engineering in engineering education was pioneered two decades ago by a group of research and academic leaders,^97−99^ WASH can and should be included in modern environmental engineering programs. Finally, the potential for global networking could be achieved via collaboration with organizations such as the Latin-American-based Inter-American Association of Sanitary and Environmental Engineering (AIDIS) which supports webinars and online and in-person courses in the area of WASH.

## 5 Implementation, Partnerships, and Engagement of Stakeholders and Community Groups

The interplay and relationship between various stakeholders (owners, funders, and service providers) are key determinants of an AD project’s success or failure. Kalina et al.^48^ identified the owner as the most important stakeholder and highlighted the owner’s ability and willingness to engage and understand AD technologies as a key indicator of project success. Involving community members through various phases of project planning and execution, considering their specific needs, and using local labor and materials, result in better uptake of the installed facilities and contribute to a sense of ownership.^8,100−104^ Specific needs include not just the physical context of space and topography, but also the preferences, concerns, constraints, and capabilities of a community. Such demand-based, rather than supply-based systems, where communities commit to partnering have been noted as drivers for success in any sanitation project.^105^

For researchers focused on the implementation of AD and sanitation in decentralized communities, it is crucial to have nontechnical experts on the team (e.g., social scientists, anthropologists).^100−102,104^ Because researchers and technology developers need to work closely with communities, they need to be aware of best practices in participatory and “community-engaged” research,^106^ including involving communities and users of AD technology in defining and prioritizing problems, as well as codesigning solutions. Building three-way trust between partner NGOs, the user community, and researchers is needed. A key is being transparent with expectations and describing not only areas of mutual benefits but also risks and unknowns. These approaches include social dimensions engineers and researchers may not always be familiar with, hence working with social scientists becomes crucial.^102^

Finally, building an ecosystem of partners from governments, the private sector, NGOs, funding agencies, and user communities is crucial to the success of an AD sanitation project. A whole set of implementation issues, such as choice of collection and transport, scale of implementation, operation and maintenance, user education, project financing, and workforce development, among others, need to be addressed. Maximizing profit as the primary goal may not be compatible with many AD sanitation projects; private companies involved would likely have a service or social business orientation that aligns with the sanitation goals of SDG6. Here, standardization and international certification of nonsewered sanitation solutions such as AD technologies and products may help with private sector involvement, as standards and certification reduce liability risks, enhance communication and innovation, and make assessments efficient.^107^ Recently developed voluntary product standards for nonsewered sanitation, ISO 30500, and ISO 31800, are seen as tightly connected to technical innovations and sustainability aspects of sanitation solutions,^108^ and AD systems and products would benefit from following these standards.

Research funding agencies understanding the need for community engagement also encourage AD sanitation researchers to appreciate the real needs of the community and identify the most appropriate context-specific solutions. Addressing the major hurdles in the “enabling environment”, technology, economic opportunities and incentives, culture, and behavior^8^ are crucial to the successful implementation of AD sanitation projects, and different contexts would require careful analysis and decision-making.

## 6 Implications

Despite the advantages and potential positive impacts of AD in sanitation, the record of decentralized AD projects in LICs and LMICs is spotty, apart from the application of AD for the treatment of municipal sewage in tropical areas, like in Southern America^109^ and municipal sludge at centralized wastewater treatment plants. Along with examples of successful AD projects in sanitation described above are hundreds of failed and abandoned biogas projects, particularly in Africa.^48^ Research shows there is no one-size-fits-all technology or management approach for delivering unmet water and sanitation needs in underserved communities.^8,110,111^ Accordingly, appropriate solutions must be context-specific^112,113^ and should be codeveloped with partner communities. These context-specific factors that are crucial to success include sustainability aspects such as acceptance, affordability, and complexity.^114^ As with any technological solution, AD projects will only be successful insofar as they are implemented along with the relevant regulatory environment, training in construction, operation and maintenance, supply chain availability, and support and commitment from those within the household and community. Addressing these issues can propel AD as a forefront solution to decentralized sanitation challenges in urban and rural communities around the world.
